# Anti-tubercular activity of a natural stilbene and its synthetic derivatives

**DOI:** 10.3205/id000036

**Published:** 2018-02-01

**Authors:** Claudia Reinheimer, Dominik Büttner, Eugen Proschak, Helge B. Bode, Volkhard A. J. Kempf, Thomas A. Wichelhaus

**Affiliations:** 1 Institute of Medical Microbiology and Infection Control, Hospital of Goethe-University, Frankfurt, Germany; 2 Institute of Pharmaceutical Chemistry, Goethe-University, Frankfurt, Germany; 3 Merck endowed chair for Molecular Biotechnology, Department of Biosciences and Buchmann Institute for Molecular Life Sciences (BMLS), Goethe-University, Frankfurt, Germany

**Keywords:** tuberculosis, drug resistance, new substances, epoxide hydrolases, stilbene

## Abstract

**Objectives:** Tuberculosis (TB) and multidrug- and extensively drug-resistant TB in particular are remaining a major global health challenge and efficient new drugs against TB are needed. This study evaluated the anti-tubercular activity of a natural stilbene and its synthetic derivatives against *M. tuberculosis*.

**Methods:** Isopropylstilbene and its synthetic derivatives were analyzed for their anti-tubercular activity against *M. tuberculosis* ATCC 27294 as well as multidrug- and extensively drug-resistant *M. tuberculosis* clinical isolates by using MGIT 960 instrumentation and EpiCenter software equipped with TB eXiST module. Cytotoxic effects of drug candidates were determined by a MTT dye reduction assay using A549 adenocarcinomic human alveolar basal epithelial cells.

**Results:** Growth of *M. tuberculosis* ATCC 27294 was suppressed by the natural isopropylstilbene HB64 as well as synthetic derivatives DB56 and DB55 at 25 µg/ml. Growth of clinical isolates MDR and XDR *M. tuberculosis* was suppressed by HB64 at 100 µg/ml as well as by synthetic derivatives DB56 and DB55 at 50 µg/ml and 25 µg/ml, respectively. No anti-tubercular activity was demonstrated for synthetic derivatives DB53, EB251, and RB57 at 100 µg/ml. Toxicity in terms of IC_50_ values of HB64, DB55 and DB56 were 7.92 µg/ml, 12.15 µg/ml and 16.01 µg/ml, respectively.

**Conclusions:** Synthetical derivatives of stilbene might be effective candidates as anti-tubercular drugs. However, toxicity of these substances as determined by IC_50_ values might limit therapeutic success *in vivo*. Further investigations should address lowering the toxicity for parenteral administration by remodeling stilbene derivatives.

## Introduction

Tuberculosis (TB) incidence is declining globally, however, multidrug-resistant TB (MDR-TB), defined as TB with resistance to isoniazid (INH) and rifampicin (R), and extensively drug-resistant (XDR-TB), defined as MDR with additional resistance to a fluoroquinolone and to one or more of the injectable drugs, remain major global health challenges [1]. MDR-TB strains are representing a major threat to TB control since the 1990s, and the emergence of the first XDR-TB cases in 2006 [2] has impressively demonstrated that treatment options for XDR-TB have become alarmingly narrow and the need for new drugs is pressing. While evidence supports the repurposing of antibiotics approved for other indications, such as linezolid [3], the new drugs bedaquiline and delamanid have recently been approved for the treatment of drug-resistant tuberculosis [4]. Despite this silver lining, the increasing burden of XDR-TB underlines the demand on efficient new substances. 

Since the sequence of the *M. tuberculosis* genome has been published, a number of characteristics have been described, *e.g.* the genome encodes for at least 20 epoxide hydrolases (EHs), which has formerly been reported by Cole et al. to be an unusually large number for a single bacterium [5]. EHs are key enzymes in the arachidonic acid cascade [6] and essential to many organisms because of their ability to transform chemically reactive and detrimental epoxides into less reactive substances [7], [8]. 

Substances to inhibit EHs may therefore promising substances in future anti-tubercular therapy. 

Stilbenes have formerly been observed to have an inhibitory effect on EHs [9]. Natural stilbenes are a group of nonflavonoid phytochemicals of polyphenolic structure characterized by the presence of a 1,2-diphenylethylene core [10], [11]. Considering (a) the stilbenes’ inhibitory effect on EHs, (b) the potential toxicity of EH substrates to *M. tuberculosis* in general and (c) the unusual large number of EHs in *M. tuberculosis*, EHs therefore are proposed to be a promising target for innovative anti-tubercular drugs (ATD). The role of stilbenes and stilbene-derived substances as potentially innovative ATD has scientifically not been addressed yet, to our knowledge. This study therefore aims at evaluation the anti-tubercular activity of a natural stilbene and its synthetic derivatives against *M. tuberculosis*.

## Material and methods

### Natural stilbenes and its synthetic derivatives

**Figure 1:**
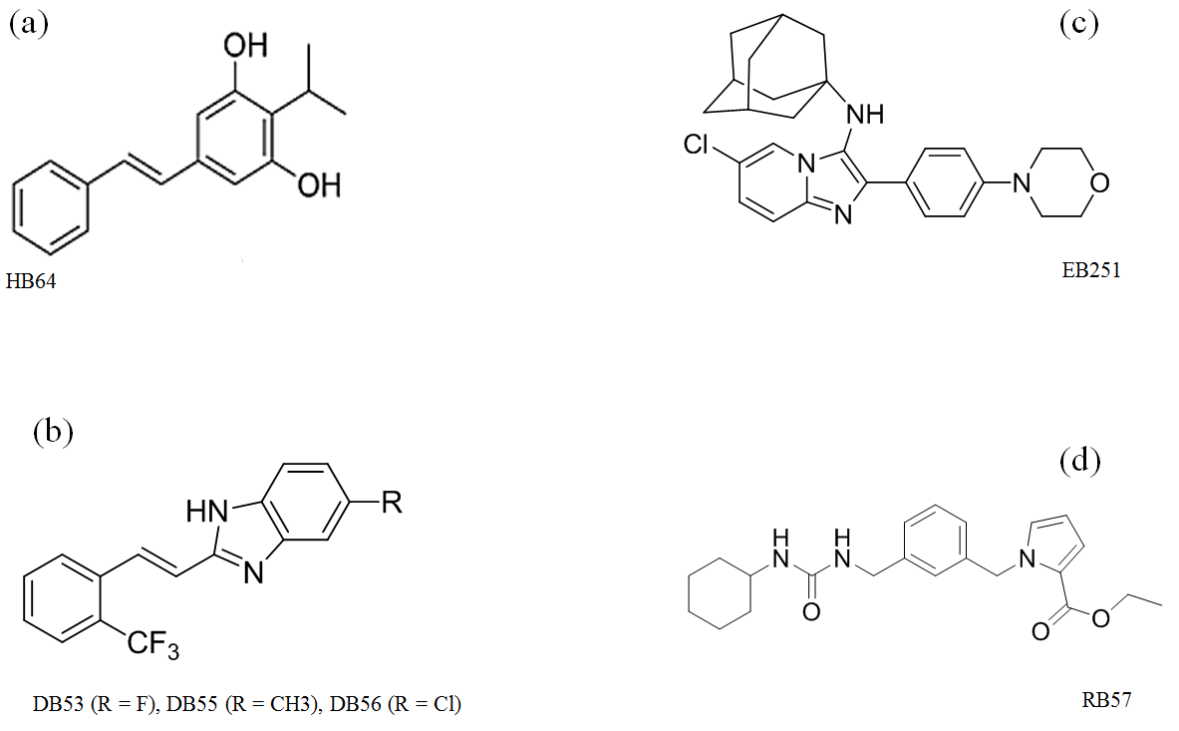
Natural stilbene and its synthetic derivatives evaluated in this study a) Natural stilbene, isopropylstilbene (HB64); b) (E)-styryl-1H-benzo[d]imidazoles with DB53 (R=F), DB55 (R=CH3), DB56 (R=Cl); c) N-((3s, 5s, 7s)-adamantam-1-yl)-6-chloro-2-(4-morpholinophenyl)imidazo[1, 2-a]pyridine-3-amine (EB251); d) 1-(3-((3-cyclohexylureido)methyl)benzyl)-1H-pyrrole-2-carboxylate (RB57)

The natural stilbene isopropylstilbene (HB64, Figure 1a (Fig. 1)) isolated from *Photorhabdus luminescens* [12], [13] as well as its synthetic derivatives were analyzed for their anti-tubercular activity.

#### (E)-styryl-1H-benzo[d]imidazoles (DB53, DB55, DB56)

In a round-bottom flask 250 mg of 3,4-diaminotoluene derivatives (1.0 eq), o-trifluoromethyl cinnamic acid (1.0 eq) and EDC*HCl (1.5 eq) was dissolved in 10 ml DMF. Catalytic amounts of DMAP (0.05 eq) and imidazole (0.05 eq) were added, and the reaction was stirred for 4 h at room temperature. After addition of 150 ml of distilled water, the precipitated solid was obtained by vacuum filtration and washed twice with 10 ml of water.

100 mg of cinnamic acid amides were suspended in 2 ml of 6 M aqueous hydrochloric acid. 2.5 ml of EtOH were added, and the mixture was heated under microwave irradiation for 10 minutes at 120°C. The reaction was cooled to room temperature, and subsequently cooled at 4°C. The resulting sediment was filtered off and washed with water. Substances are named DB53 (R=F), DB55 (R=CH_3_), DB56 (R=Cl) (Figure 1b (Fig. 1)).

#### N-((3s, 5s, 7s)-adamantam-1-yl)-6-chloro-2-(4-morpholinophenyl)imidazo[1, 2-a]pyridine-3-amine (EB251) and ethyl 1-(3-((3-cyclohexylureido)methyl)benzyl)-1H-pyrrole-2-carboxylate (RB57)

Synthesis has formerly been described by Buscató et al. [14], [15] (Figure 1c,d (Fig. 1)).

### Bacterial strains

Reference strain *M. tuberculosis* ATCC 27294 as well as MDR and XDR *M. tuberculosis* clinical isolates were used to determine the anti-tubercular activity of a natural stilbene and its synthetical derivatives. 

### Susceptibility testing of M. tuberculosis 

The MGIT 960 system was used for anti-tubercular susceptibility testing as previously described [16]. MGIT tubes supplemented with 0.8 ml of supplement (MGIT 960 SIRE supplement; Becton Dickinson) were inoculated with 0.1 ml of the drug solution at various concentrations and 0.5 ml of the test strain suspension, i.e. *M. tuberculosis* ATCC 27294 as well as MDR and XDR *M. tuberculosis* clinical isolates. For preparation of the drug-free growth control tube, the organism suspension was diluted 1:100 with sterile saline, and then 0.5 ml was inoculated into the tube (proportion testing). Quantitative drug susceptibility testing (DST) was performed using the MGIT 960 instrumentation and EpiCenter software version 5.53 equipped with the TB eXiST module (Becton Dickinson), providing features as previously described [16], including automated recording of the readings, additional incubation time beyond the time to positivity of the drug-free control, and graphical representation of the growth unit (GU) value increase. The susceptibility testing sets were placed in the MGIT 960 instrument and continuously monitored using EpiCenter TB eXiST software.

The susceptibility testing was performed in triplicate. Results were interpreted as follows: at the time when the growth unit (GU) of the drug-free control tube was >400, if the GU of the drug-containing tube to be compared was ≥100, the strain was resistant (R). If the GU of the drug-containing tube was <100, it was interpreted as susceptible (S). Rifampicin and isoniazid were used as positive control drugs.

### Assessment of cytotoxic effects

Cytotoxic effects of drug candidates were determined by a 3-(4,5-dimethylthiazol-2-yl)-2,5-diphenyltetrazolium bromide (MTT) dye reduction assay. Cells were plated in 96-well microtiter plates at a density 20,000–50,000 cells/ well [17]. The cells were incubated in a culture medium at concentrations between 0.19 and 100 µg/ml of the anti-tubercular drug. After 5 days of incubation at 37.0°C in 5% CO_2_ atmosphere, 25 µl MTT substrate (2 mg/ml) was added and plates were incubated at 37.0°C for 4 hours. After incubation, cells were lysed in 100 ml buffer containing 2% (w/v) SDS and 50% (v/v) *N,N*-dimethylformamide with the pH adjusted to 4.7. Absorbance at 560/620 nm was determined for each well using a 96-well multiscanner. After correcting for the background, the results were expressed as percentage viability relative to a control culture which received no drug.

## Results and discussion

The global spread of TB and its multi- or extensively drug resistant strains is remaining a matter of public health concern worldwide [1], [2]. Therefore, new compounds against *M. tuberculosis* are urgently needed [18]. In order to address this issue, we tested the anti-tubercular activity of a natural stilbene and its synthetic derivatives.

Numerous assay systems have been established for the evaluation of compounds against *M. tuberculosis* and we followed a method based on monitoring growth/inhibition via fluorometric detection of oxygen consumption [16].

A total of six substances were used for quantitative DST on the basis of the MGIT 960 instrumentation and EpiCenter. Growth of *M. tuberculosis* ATCC 27294 was suppressed by HB64 at 25 µg/ml as well as synthetic derivatives DB56 and DB55 at 25 µg/ml each (Table 1 (Tab. 1)). Growth of clinical isolate MDR *M. tuberculosis* was suppressed by HB64 at 100 µg/ml as well as by synthetic derivatives DB56 and DB55 at 50 µg/ml and 25 µg/ml, respectively (Table 1 (Tab. 1)). Furthermore, HB64, DB55 and DB56 were tested against clinical isolate XDR *M. tuberculosis* with growth suppression at 100 µg/ml, 25 µg/ml and 50 µg/ml, respectively (Table 1 (Tab. 1)). Interestingly, compared with the natural stilbene, synthetic derivatives (DB55 and DB56) showed enhanced *in vitro* activity against MDR and XDR *M. tuberculosis*.

The natural stilbene (HB64) also known as benvitimod has been accepted for clinical trial for the treatment of psoriasis. Benvitimod demonstrated to be an effective topical anti-inflammatory molecule that showed a negligible absorption and was well tolerated [19]. In the present study we determined the toxicity in terms of IC_50_ values for those substances that show anti-tubercular activity (Table 2 (Tab. 2)). The IC_50_ values turned out to be lower than the MIC of those substances exhibiting anti-tubercular activity, an issue that would limit systemic application *in vivo*. Furthermore, rapid metabolization of stilbenes after administration has been described [20], which might further influence pharmacokinetics and -activities.

Finally, synthetical derivatives of stilbene might be effective candidates as anti-tubercular drugs. However, toxicity of these substances as determined by IC_50_ values limit therapeutic success *in vivo*. Further investigations need to address lowering the toxicity by remodeling stilbene derivatives.

## Notes

### Competing interests

The authors declare that they have no competing interests.

### Acknowledgments

We thank Denia Frank and Kuflom Gebreamlack for excellent technical support.

### Authors’ contributions

- Conceptualization: CR, DB, EP, HB, VK, TW
- Formal analysis: CR, DB, EP, HB, VK, TW
- Funding acquisition: CR, TW
- Investigation: CR, DB, EP, HB, VK, TW
- Methodology: CR, DB, EP, HB, VK, TW
- Project administration: CR, DB, EP, HB, VK, TW
- Resources: CR, DB, EP, HB, VK, TW
- Software: CR, DB, EP, HB, VK, TW
- Supervision: CR, DB, EP, HB, VK, TW
- Validation: CR, DB, EP, HB, VK, TW
- Visualization: CR, DB, EP, HB, VK, TW
- Writing – original draft: CR, TW
- Writing – review & editing: CR, DB, EP, HB, VK, TW

### Funding

This work was supported by the Foundation *Nachlässe Marie Christine Held und Erika Hecker,* Hospital of Goethe-University, Frankfurt am Main, Germany.

## Figures and Tables

**Table 1:**
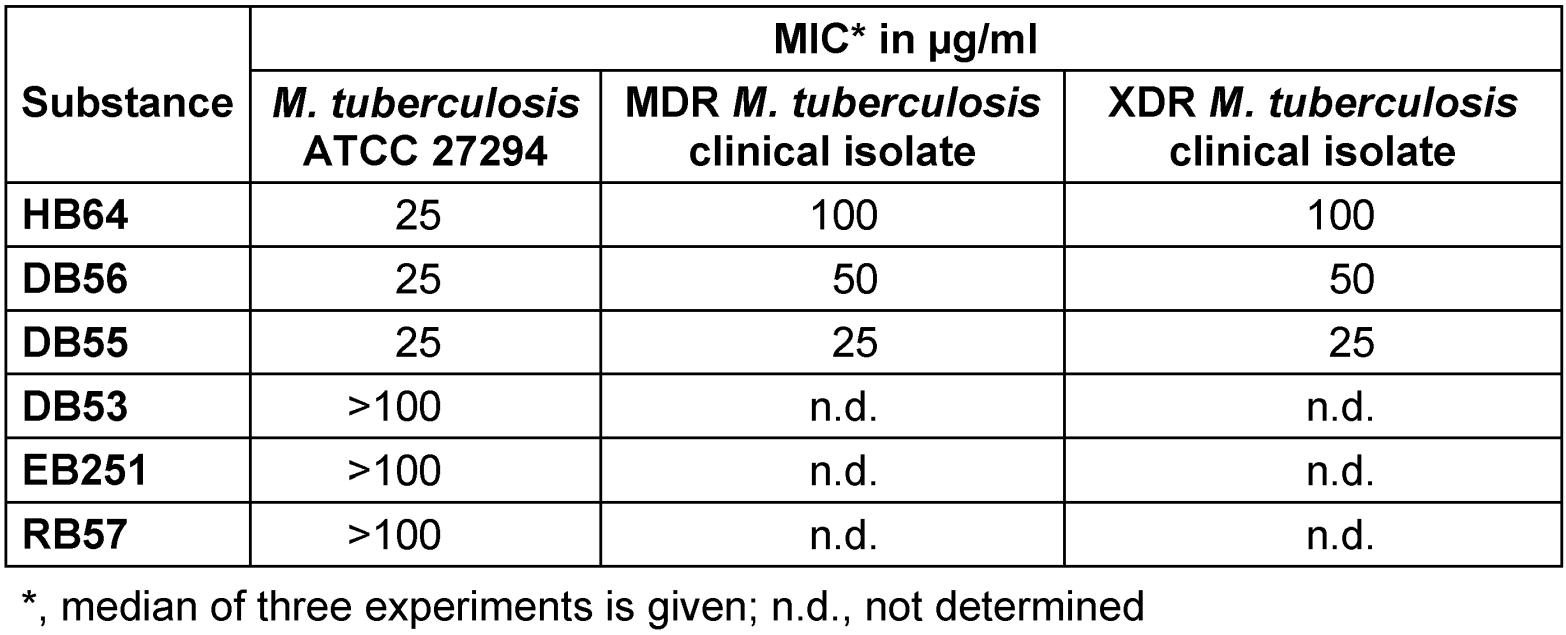
Anti-tubercular activity of stilbenes MIC of substances against *M. tuberculosis* ATCC 27294 as well as MDR and XDR *M. tuberculosis* clinical isolates

**Table 2:**
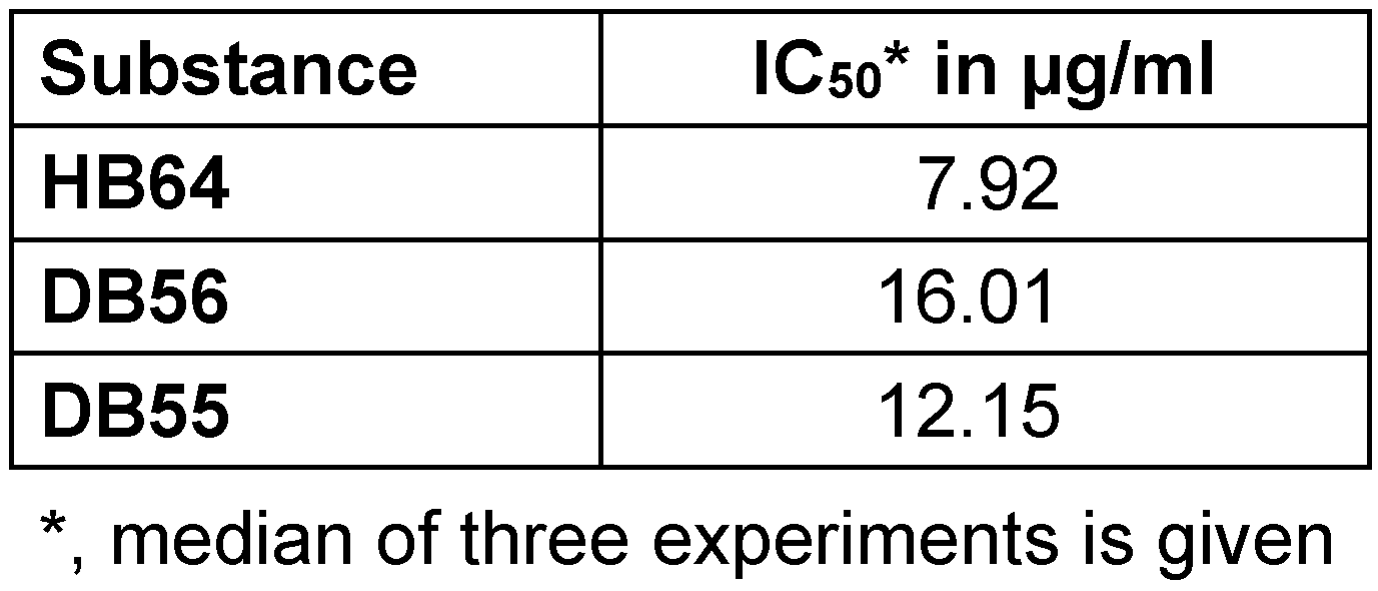
Toxicity of stilbenes IC_50_ of selected substances
